# Degree of food processing and association with overweight and abdominal obesity in adolescents

**DOI:** 10.31744/einstein_journal/2022AO6619

**Published:** 2022-05-06

**Authors:** Sineide Freitas de Souza, Maria Ester Pereira da Conceição-Machado, Priscila Ribas de Farias Costa, Carla de Magalhães Cunha, Valterlinda Alves de Oliveira Queiroz, Mônica Leila Portela de Santana, Luana de Oliveira Leite, Ana Marlúcia de Oliveira Assis

**Affiliations:** 1 School of Nutrition Universidade Federal da Bahia Salvador BA Brazil Graduate Program in Foods, Nutrition and Health, School of Nutrition, Universidade Federal da Bahia, Salvador, BA, Brazil.

**Keywords:** Adolescent, Eating, Industrialized foods, Overweight, Obesity, abdominal

## Abstract

**Objective:**

To evaluate the association between the degree of food processing, overweight, and abdominal obesity in adolescents.

**Methods:**

This is a cross-sectional study, with 576 adolescents aged 10 to 17 years, of both sexes. Food consumption was collected using the Food Frequency Questionnaire and foods classified as *in natura* or minimally processed, processed foods associated with culinary ingredients, and ultraprocessed foods. Sociodemographic data, body mass index, waist circumference and waist-to-height ratio were collected. The analysis was evaluated by the Mann-Whitney test and prevalence ratio with 95% confidence interval, considering p<0.05.

**Results:**

An intake above the third quartile of processed foods associated with culinary ingredients (prevalence ratio of 1.64; 95%CI: 1.12-2.42) and ultraprocessed (prevalence ratio of 1.58; 95%CI: 1.07-2.34) was associated with a higher prevalence of overweight. Consumption above the third quartile of ultraprocessed foods was associated with a higher prevalence of abdominal obesity, assessed by waist circumference (prevalence ratio of 2.48; 95%CI: 1.41-4.36), and waist-height ratio (prevalence ratio of 2.09; 95%CI: 1.11-3.92).

**Conclusion:**

A higher consumption of processed foods associated with culinary ingredients was related to being overweight, and ultraprocessed foods with overweight and abdominal obesity.

## INTRODUCTION

It is expected that by 2025, worldwide, there will be 206 million children and adolescents aged between 5 and 19 years with obesity, mainly in low- and middle-income countries.^(1)^ In Latin America, childhood obesity rates are among the highest in the world, with one in five children and adolescents overweight or obese.^(2)^In Brazil, according to data from the 2019 National Health Survey, 19.4% of adolescents were overweight and 6.4% were obese.^(3)^

The growing prevalence of excessive weight in childhood is of epidemiological importance because of its association with morbidity and mortality in later life -especially its association with cardiovascular disease, diabetes mellitus, musculoskeletal diseases, and some types of cancer, comprising an important economic burden for the country’s public health.^(4)^

Adolescence is a phase of intense physical and psychosocial development, marked by transformations and commonly characterized by breaking behavioral patterns, detaching from family eating habits, imitating the habits of the social group in which the adolescent wishes to be inserted, and a strong influence of the media.^(5)^ These behaviors may be associated with what is observed in adolescent food consumption, characterized by a high ingestion of cookies, candies, pasta, and sugary drinks, which are highly processed foods.^(6)^

Foods with high degree of processing have high calorie density and high sodium, saturated fat, and simple carbohydrate content, being associated with weight gain. In addition, they are increased with additives, such as flavor enhancers, which encourage excessive consumption due to hyper-palatability, as well as preservatives, dyes, and other substances associated with health problems.^(7,8)^ Thus, the high degree of food processing may be one of the factors contributing to the increased occurrence of obesity.^(9)^

Studies that evaluate food consumption, especially related to the degree of processing in childhood and adolescence, as well as its influence on nutritional status, are important to contribute to public policies that are more effective to prevent overweight and associated comorbidities. Considering that adolescence is a period of transformation, this also makes it an opportunity to establish and solidify healthy eating habits.

## OBJECTIVE

To evaluate the association between food consumption, considering the degree of food processing, overweight, and abdominal obesity in adolescents from public schools.

## METHODS

This is a cross-sectional study, conducted with baseline data from a cohort entitled “School and family environment and cardiovascular risk: a prospective approach”, which, in turn, is part of a larger study called “School policy and cardiovascular risk: a multi-country study”, carried out in four countries: England, Brazil, India, and the United States. In Brazil, the investigation included adolescents of both sexes, aged between 10 and 17 years, from five public schools – in that, two were military and three state schools, from different neighborhoods of the city of Salvador (State of Bahia - BA). The choice of schools was made by convenience, based on the interest of managers in participating in the project.

We used information from 576 adolescents, who presented all data of interest for this investigation. With this sample size, this study had 80%, 82%, and 92% power to identify overweight, obesity, and abdominal obesity, respectively, among individuals who had high and low consumption of ultraprocessed foods.^(7)^

Pregnancy status, lactation, and physical disabilities that prevented anthropometric evaluation were adopted as non-inclusion criteria.

The study was approved by the Ethics and Research Committee of the *Escola de Nutrição da Universidade Federal da Bahia* (UFBA), # 1.139.343, and CAAE: 42053014.0.0000.5023.

### Data collection and definition of variables

The research team invited the students, explained the objectives of the study, and handed out the questionnaires for completion. Those who agreed to participate signed the Assent Form and presented the Informed Consent Form duly signed by their parents or guardians.

Data collection occurred in 2017 and was performed by a properly trained team, during which time 619 completed questionnaires were returned, and were reviewed by the research team as soon as they arrived from the field. After checking the collected data, they were coded and double entered, avoiding typing errors, using the EpiData software, version 3.1.

Sociodemographic information was recorded by parents or guardians in a structured questionnaire sent to the home.

The economic status was obtained according to the criteria of the Brazilian Association of Research Companies (ABEP), which classifies the results into six socioeconomic strata (A, B1, B2, C1, C2, D-E), taking into account consumer goods, household characteristics, and the level of education of the head of the household, which correlate with the average household income.^(10)^ Socioeconomic strata were categorized into higher (A+B1+B2) and lower (C1+C2+D-E).

The information on habitual food consumption in the previous 6 months was obtained by the Food Frequency Questionnaire (FFQ) validated for this population,^(11)^ and applied by properly trained researchers. This instrument was composed of 99 foods and preparations, whose frequency of recording included the options: never/rarely; one to three times a month; once a week; two to four times a week; and more than four times a week. Subsequently, the food consumption information was categorized into four groups, adopting the Nova^(12)^ classification that characterizes foods according to the nature and degree of processing: *in natura* or minimally processed foods, culinary ingredients, processed foods, and ultraprocessed foods. For analysis purposes, the groups culinary ingredients and processed foods were grouped together, totaling three groups of foods with different degrees of processing. The lists of foods that made up the groups are presented on table 1.

**Table 1:** List of foods consumed, according to the degree of processing

Food groups	Description
*In natura* and minimally processed foods (G1)	
Fruit and vegetables	Fruits; leafy/cooked vegetables; legumes and oilseeds
Meats and organs/eggs	Cooked/baked chicken, fish, and liver; cooked seafood; beef; eggs
Milk	Whole and skimmed
Roots and tubers	Manioc, sweet potato, and plantain
Cereals and flour/meal	Oats, wheat germ; cassava flour, corn flour, couscous, and homemade popcorn
Rice and pasta	Rice, white and whole wheat noodles
Leguminous plants	*Carioquinha* beans, green beans, and other varieties
Fruit juices and coffee	Fruit juice and fruit pulp; coffee
Processed foods and culinary ingredients (G2)	
Bread	White/whole wheat bread
Sweets/ homemade cakes	Homemade sweets with only sugar and natural ingredients; popsicles, fruit ice creams; homemade cakes
Salted meat	Jerked beef, sun-dried meat
Preparation with beans without sausages	*Acarajé; abará*
Culinary ingredients	Sugar, salt, oil, olive and palm oils, coconut milk
Cheese	White and yellow
Ultraprocessed foods (G3)	
Sugar-sweetened beverages	Chocolate, soft drinks, carbonated drinks, artificial juices, ice cream, creamsicles, and energy drinks
Industrialized sweets	Candies, cream-filled cookies, industrialized candies (guava paste and marmalade), ready-made cakes, chewing gum, lollipops, chocolates, jellies, and creamy ice cream
Flours	Industrialized cereal flours
Dairy products	Cream cheese, flavored dairy/yogurt drinks
Snacks and pastas	Fast-food, fried and baked snacks, packaged snacks, potato chips, pizza, lasagna, hot-dogs, sandwiches, French fries, industrialized popcorn, cereal bars, cookies
Sauces and butter	Ketchup, mayonnaise, pizza sauce, and margarine/butter
Processed meats and preparations with processed meats and sausages	*Feijoada, feijão tropeiro, dobradinha, calabresa*, wieners, and sausages in general
Instant preparations	Ready-made soups, instant noodles

To quantify the intake of food from the groups, the frequency of consumption of each food was converted into daily consumption, as proposed by Coelho.^(13)^ This estimate is calculated from the frequency (weekly or monthly) of consumption informed in the FFQ and the information on how many times the food was consumed during the day. For the estimates of weekly and monthly consumption, the mean consumption was used, divided by the period - weekly by seven, monthly by 30 - and multiplied by the previously established standardized portion of the food. If the food was consumed more than once a day, the final value obtained was multiplied by the number of times consumed per day. Subsequently, the values found for each food of the three food groups were added up, determining the total grams of each food group consumed per day by the adolescent.

Regarding anthropometric data, the adolescents’ weight was measured by a trained staff and obtained using a Filizola^®^ portable digital scale, with a capacity of 150kg and accuracy of 100g. The adolescents were barefoot, wearing school uniforms, and positioned on the scale platform with body weight equally distributed between the feet. Height was measured using a Leicester Height Measure stadiometer, reading to the nearest millimeter. The adolescents were measured barefoot, with no head adornments, positioned vertically with arms extended along the body, shoulders relaxed, heels together, and head positioned on the Frankfurt plane. The height and weight procedures were performed in duplicate by two different raters, and the average between the two measurements was adopted as the final measurement. The variation allowed between the two measurements was 0.1kg for weight and 0.1cm for height.^(14)^

Waist circumference was measured with the teenagers standing upright, weight evenly distributed on both feet, and arms along the body. The inelastic, fiberglass, centimeter scale tape measure was positioned at the midpoint between the last rib and the superior iliac crest. The tape was adjusted to the body in such a way as to avoid looseness or compression of the skin. The reading was taken at the time of expiration, with the individuals breathing smoothly, and taken to the nearest millimeter of the scale. If the variation between the evaluators was greater than 0.5cm, a new measurement was taken, and the average of the two closest measurements was adopted.^(14)^

Body mass index by age (BMI/A) was assessed using the curves by sex recommended by the World Health Organization (WHO) for individuals aged 5 to 19 years,^(15)^ and adolescents with BMI/A above the 85^th^ percentile were considered overweight. The waist circumference was used as an indicator of abdominal obesity, considering the 80^th^ percentile, according to cutoff points by sex and age proposed by Taylor et al.^(16)^ The waist-to-height ratio (WHtR) was obtained by dividing the waist circumference (cm) by the height (cm). Abdominal obesity was also assessed, considering a value greater than or equal to 0.50.^(17)^

### Statistical analysis

For data evaluation, descriptive analysis was used by means of mean and standard deviation, or median and interquartile range (IQR) for continuous variables, and absolute and relative frequencies, for categorical variables. The amount in grams of each food group consumed per day by the adolescents was obtained by calculating the median and IQR. It was established as high food consumption when the intake was above the third quartile of the sample. Differences between quantitative variables were assessed by the Mann-Whitney test. To identify the association between anthropometric variables and food intake, Poisson multiple regression models (which belong to the generalized linear models family) were used via an estimator, the prevalence ratio (PR), which compares the prevalence of the outcome in exposed individuals with the prevalence in non-exposed individuals.^(18)^ According to Kleinbaum,^(18)^ if the assumption of “rare disease” (arbitrarily established as a prevalence less than 10%) cannot be reached for a certain outcome, the calculation of the odds ratio (OR) is inadvisable, because it tends to present point estimates that are overestimated, with 95% confidence intervals (95%CI) that are less accurate.

Initially, bivariate regression was performed, and the variables that presented p value ≤0.20 entered the multivariate model. Only variables with p value <0.05 remained in the final model. To evaluate the fit of Poisson regression models, Akaike’s information criterion was used.^(19)^ It is a measure of quality of fit of a statistical model estimated based on the relative measure of information lost in the adoption of a given model. The less information lost, the better the model fit (*i.e*., the lower the Akaike’s information criterion, the better the fit).

All statistical analyses were performed in Stata^®^ version 16.0.

## RESULTS

The sample consisted of 576 adolescents, with a median age of 13.30 years (12.11 to 14.6). Most participants were female (65.5%), aged under 14 years (63.5%), and from the lowest economic category (59.4%). Regarding anthropometric status, most adolescents were eutrophic (65.3%), but overweight was identified in 31.2% (overweight in 15.6% and obesity in 15.6%). Regarding abdominal obesity, 22.9% had an increased waist circumference, and 16.8% had a high WHtR (Table 2).

**Table 2:** Demographic, economic, and anthropometric characteristics of adolescents from public schools

Variables	n (٪)
Sex	
Male	199 (34.5)
Female	377 (65.5)
Age, years	
<14	366 (63.5)
≥14	210 (36.5)
Economic category*	
Higher (A+B)	171 (40.6)
Lower (C+D+E)	250 (59.4)
Anthropometric status	
BMI/A	
Malnutrition	20 (3.5)
Eutrophy	376 (65.3)
Overweight	90 (15.6)
Obesity	90 (15.6)
Abdominal obesity	
Waist circumference	
Increased	132 (22.9)
Adequate	444 (77.1)
WHtR	
Increased	97 (16.8)
Adequate	479 (83.2)

* n=421.

BMI/A: body mass index by age; WHtR: waist-to-height ratio.

Regarding food consumption, it was found that adolescents with no overweight had a significantly higher consumption of processed foods and culinary ingredients (410.3 *versus* 354.5g; p=0.001) and ultraprocessed foods (975.5 *versus* 778.9g; p=0.001) than adolescents with overweight. We also observed adolescents without excess abdominal fat consumed significantly more ultraprocessed foods than those with abdominal obesity, as assessed by both indicators (waist circumference: 992.1 *versus* 732.4g; p<0.001; WHtR: 932.0 *versus* 777.6g; p=0.012) (Table 3).

**Table 3:** Food consumption (in grams) as per the degree of food processing of adolescents by body mass index by age, waist circumference, and waist-to-height ratio

Degree of food processing (g/day)	No excess/adequate Median (IQR)	With excess/increased Median (IQR)	p value*
BMI/A			
*In natura* and minimally processed	1,544.2 (1,105.8-2,161.5)	1,442.9 (937.5-1,988.9)	0.062
Processed foods and culinary ingredients	410.3 (280.2-578.0)	354.5 (235.9-490.9)	0.001
Ultraprocessed	975.5 (601.0-1,641.6)	778.9 (462.4-1,311.3)	0.001
Waist circumference			
*In natura* and minimally processed	1,545.7 (1,095.8-2,157.2)	1,394.5 (963.2-1,975.2)	0.061
Processed foods and culinary ingredients	401.7 (271.1-562.4)	368.6 (258.4-500.0)	0.107
Ultraprocessed	992.1 (602.5-1,640.3)	732.4 (448.0-1,178.6)	<0.001
WHtR			
*In natura* and minimally processed	1,544.8 (1,088.2-2,154.9)	1,394.0 (955.5-1,911.5)	0.076
Processed foods and culinary ingredients	398.6 (268.6-560.2)	382.6 (266.1-503.0)	0.384
Ultraprocessed	932.0 (572.0-1,612.5)	777.6 (467.0-1,280.0)	0.012

* Mann-Whitney test.

IQR: interquartile range; BMI/A: body mass index by age; WHtR: waist-to-height ratio.

Adolescents with consumption above the third quartile of processed foods and culinary ingredients had an overweight prevalence 1.64 times higher, when compared to those who consumed below this quartile (PR of 1.64; 95%CI: 1.12-2.42). As for the consumption of ultraprocessed food, the participants who consumed this group of food above the third quartile had a prevalence of overweight 1.58 times higher, when compared to those who consumed below this quartile (PR of 1.58; 95%CI: 1.07-2.34). Considering abdominal obesity, adolescents who consumed ultraprocessed foods above the third quartile had 2.48 times higher prevalence of increased waist circumference (PR of 2.48; 95%CI: 1.41-4.36) and 2.09 times higher prevalence of high WHtR compared to those below this quartile (PR of 2.09; 95%CI: 1.11-3.92). These results were adjusted for sex, age, and socioeconomic status (Table 4).

**Table 4:** Prevalence ratio between overweight and abdominal obesity as per food consumption, by the degree of food processing, demographic, and economic variables of adolescents

Variables	PR_bivariate_ (95%CI)	PR_multivariate_ (95%CI)*
	Excess weight	
Sex		
Male	1	
Female	1.07 (0.78-1.45)	
Age, years		
<14	1	
≥14	0.89 (0.65-1.21)	
Economic category		
Lower (C+ D + E)	1	
Higher (A + B)	1.00 (0.71-1.41)	
*In natura* and minimally processed foods		
Less than or equal to the 3^rd^ quartile	1	
Above the 3^rd^ quartile	1.17 (0.87-1.57)	1.05 (0.76-1.44)
Processed foods and culinary ingredients		
Less than or equal to the 3^rd^ quartile	1	
Above the 3^rd^ quartile	1.54 (1.10-2.15)	1.64 (1.12-2.42)
Ultraprocessed foods		
Less than or equal to the 3^rd^ quartile	1	
Above the 3^rd^ quartile	1.67 (1.18-2.35)	1.58 (1.07-2.34)
	Increased WC	
Sex		
Male	1	
Female	1.15 (0.81-1.64)	
Age, years		
<14	1	
≥14	0.81 (0.56-1.17)	
Economic category		
Lower (C+ D + E)	1	
Higher (A + B)	1.12 (0.75-1.67)	
*In natura* and minimally processed foods		
Less than or equal to the 3^rd^ quartile	1	
Above the 3^rd^ quartile	1.30 (0.89-1.89)	1.20 (0.80-1.79)
Processed foods and culinary ingredients		
Less than or equal to the 3^rd^ quartile	1	
Above the 3^rd^ quartile	1.36 (0.92-1.99)	1.35 (0.88-2.07)
Ultraprocessed foods		
Less than or equal to the 3^rd^ quartile	1	
Above the 3^rd^ quartile	2.60 (1.57-4.30)	2.48 (1.41-4.36)
	Increased WHtR	
Sex		
Male	1	
Female	1.06 (0.70-1.61)	
Age, years		
<14	1	
≥14	0.89 (0.59-1.37)	
Economic category		
Lower (C+ D + E)	1	
Higher (A + B)	1.12 (0.70-1.80)	
*In natura* and minimally processed foods		
Less than or equal to the 3^rd^ quartile	1	
Above the 3^rd^ quartile	1.28 (0.81-2.02)	1.13 (0.69-1.84)
Processed foods and culinary ingredients		
Less than or equal to the 3^rd^ quartile	1	
Above the 3^rd^ quartile	1.37 (0.86-2.18)	1.36 (0.80-2.30)
Ultraprocessed foods		
Less than or equal to the 3^rd^ quartile	1	
Above the 3^rd^ quartile	2.36 (1.33-4.19)	2.09 (1.11-3.92)

* Adjusted for age, sex, and economic category.

PR: prevalence ratio; 95%CI: 95% confidence interval; WHtR: waist-stature ratio.

## DISCUSSION

The present study recognized adolescents without excess weight had a higher consumption, in grams, of more processed foods than those who were overweight. In the regression analysis, it was identified that adolescents with consumption above the third quartile of processed foods associated with culinary ingredients, and ultraprocessed foods had a higher prevalence of overweight than those with a lower consumption of these foods. It was also found adolescents with consumption above the third quartile of ultraprocessed foods had a higher prevalence of abdominal obesity. These results corroborate the findings of other investigations.^(20-22)^

A cross-sectional study using data from 30,243 participants older than 10 years found that consumption of ultraprocessed foods was associated with higher BMI (p<0.001), and a higher prevalence of obesity (p<0.001) and overweight (p<0.02).^(21)^ A systematic review, which evaluated 26 studies of children and adolescents, 15 of them cohorts, found a positive association between consumption of ultraprocessed food groups and soft drinks, sugar-sweetened beverages and body fat, in most investigations.^(21)^

Another systematic review evaluating 11 studies of South Asian adolescents found that frequent consumption of fast food/junk food and foods with high calorie density, classified as ultraprocessed, was an individual risk factor for overweight and obesity, with a statistically significant association.^(22)^ These results agree with data published in the 2019 Indian Pediatrics guideline concluding that consumption of junk food, ultraprocessed foods, sugary and energy drinks is associated with higher BMI in children and adolescents.^(23)^

The present investigation also found that adolescents with a lower consumption of ultraprocessed foods had a lower prevalence of abdominal obesity. In the literature searched, no studies were identified that evaluated the relation between consumption of ultraprocessed foods and abdominal obesity. The study that came closest to this subject was conducted with adolescents in the United Kingdom. It evaluated the role of food environment as a determinant of nutritional status, taking into account the availability of access to establishments that sell fast food. The authors found a positive association between the number of such facilities close to the school environment, and an increase by 0.021 (95%CI: 0.007-0.033) in waist circumference,^(24)^ thus suggesting a possible relation between consumption of these foods and abdominal obesity.

Studies show a significant consumption of ultraprocessed foods in adolescence,^(6,7,25,26)^ a very concerning fact due to the association with cardiometabolic changes and other health problems. In Brazil, dietary patterns have undergone intense changes in recent years and, between 2002 and 2019, there was a significant increase in the percentage of average monthly expenditure with food away from home. At the same time, it was observed the expenses with food at home had decreased in the participation of the group of cereals, legumes, and oilseeds, classified as minimally processed foods, from 10.4% to 5.0%, while there was an increase in spending on ready-made, ultraprocessed foods, which rose from 2.3 to 3.4%.^(27)^

It is believed that the change in food consumption may be related to changes in the behavioral pattern of society, which every day requires more practicality and less time spent on food preparation at home, contributing to the increased consumption of ready or semi-ready foods. In parallel, there was a decrease in family meals, the cost of ready meals became more economical than fresh food, and ultraprocessed foods are usually highly palatable, contributing to the increase of their intake.^(28)^ High consumption of processed and ultraprocessed foods may also be associated with inadequate intake of micronutrients, as well as of protein and fiber, nutrients involved in regulatory and structural processes in the body. In addition, ultraprocessed foods may provide higher dietary content of added sugars,^(8)^ which may predispose to serious health problems, such as cardiometabolic changes, diabetes, hypertension, and premature death.^(29)^

Thus, developing more effective strategies for promoting healthier eating from childhood is important, with special attention to adolescence, a period characterized by the formation of new eating habits, in which individuals increasingly values their independence, and gradually becoming responsible for their food choices. “Eating” is also part of social relationships and, for adolescents, it starts to have a meaning of belonging to the community in which they want to be inserted. This act represents “status” and, in this process, the media have significant influence on food choices. In this sense, ultraprocessed foods have abundant media coverage in programs and digital channels aimed at adolescents, highlighting the importance of adopting relevant strategies regarding laws and regulation of advertisements, not only for early childhood, but also for the adolescence.^(25,30,31)^

It is noteworthy that several studies conducted with adolescents find an inverse association between consumption of ultraprocessed foods and overweight^(6,25,31,32)^ These results may reflect the presence of reverse causality bias,^(31)^*i.e*., overweight adolescents may have reduced the intake of less healthy foods to lose weight, and, at the time of the investigation, this change in food intake could not be detected because it was a cross-sectional study. This phenomenon may justify the findings of the evaluation by median weight intake per day.

It is also notable that standardization in the classification of foods regarding processing is important in view of the great variability of nomenclatures and definitions, especially of the ultraprocessed foods, with terminologies such as “junk food,” “fast food,” among others, making the comparison among studies difficult. The Nova^(12,33)^ classification adopted in this study, has been proposed as an alternative to a standardized classification among studies.

Regarding the limitations of the study, it is difficult to compare food intake with other investigations, given the wide variety of methodologies adopted. Some studies use as an estimate of habitual intake the percentage contribution of the group of foods eaten in the total calories per day, while this study used the total grams per day of the food group consumed. In addition, food consumption measured by the FFQ may not represent the amount consumed by adolescents, since it is a memory-dependent tool and has the portion of food previously established. Furthermore, we must consider that overweight adolescents may have underestimated food intake, out of shame or fear of being recriminated, a fact also reported in the literature.^(34)^

However, we highlight the use of a sample that was able to contemplate different social realities, with adolescents from various neighborhoods of the city, downtown and outskirts, as well as the methodological rigor through previous training of the team, with anthropometric data measured directly from the population investigated and the use of validated instruments. In addition, the use of robust and statistical methods appropriate for the data acquired favors the quality of the information identified here.

To broaden the understanding of the impact consumption of processed and ultraprocessed foods in the adolescent population, more robust investigations are suggested, such as cohort studies, using methods that allow the combination of different instruments to measure food intake, such as the recall, the food log, and the FFQ. These would improve the modeling probability, and quantity estimation of habitual food intake.

## CONCLUSION

Consumption above the third quartile of processed foods and culinary ingredients was associated with overweight, and consumption of ultraprocessed foods was related to excess weight and abdominal obesity.
